# Petrographic and geochemical analyses to characterise the source of built historical natural stones — a case study of the volcanic stones from historical quarries and Baoguosi Temple in the city of Ningbo, China

**DOI:** 10.1186/s43238-023-00091-3

**Published:** 2023-06-05

**Authors:** Xiuwei Guo, Yawen Zhang, Xuemin Xu, Shibing Dai

**Affiliations:** 1 School of Mechanics and Engineering Science, Shanghai University, Shanghai, 200444 China; 2 Institute for the Conservation of Cultural Heritage, Shanghai University, Shanghai, 200444 China; 3 Tianyige Museum, Ningbo, 315010 China; 4 Architectural Conservation Laboratory, College of Architecture and Urban Planning, Tongji University, Shanghai, 200092 China

**Keywords:** historical built natural stones, Meiyuanshi-stone, petrography, geochemistry, sources of historical stone

## Abstract

Characterising and sourcing natural stones are essential for not only understanding the historical information carried by heritage buildings and cultural heritage sites, but also providing necessary data for restoration and conservations. Petrographic analyses by polarised microscopy, along with the integrated chemical data acquired by inductively coupled plasma-mass spectrometry (ICP-MS) and X-ray diffraction (XRD) analysis, allowed us to ascertain the compositions of stone materials. In this paper it is applied on samples collected from quarries of “three famous stones (Meiyuanshi-, Xiaoxishi-, and Dayinshi-stone)” in Ningbo and from the Sumeru platform in the main hall of Baoguosi Temple (Ningbo, Zhejiang, China). Comparison of petrographic features, major and trace elements of the stones studied indicated that they are all tuffs but of different characteristics and origin. Moreover, we were able to confirm that the Sumeru platform in Baoguosi is made of Meiyuanshi-stone. The results have demonstrated the suitability of the approach and present a practicable solution for other stone buildings.

## Introduction

Sources of historical natural stones are interests of history, culture and architecture. The trading, art craftsmanship and contexts of prestige natural stones, like the Meiyuanshi-stone, which will be discussed in this paper, or granite and marbles used for decoration and foundations of commercial and public buildings, is part of research topics of history and architecture (Yu 2022).

**Fig. 1:**
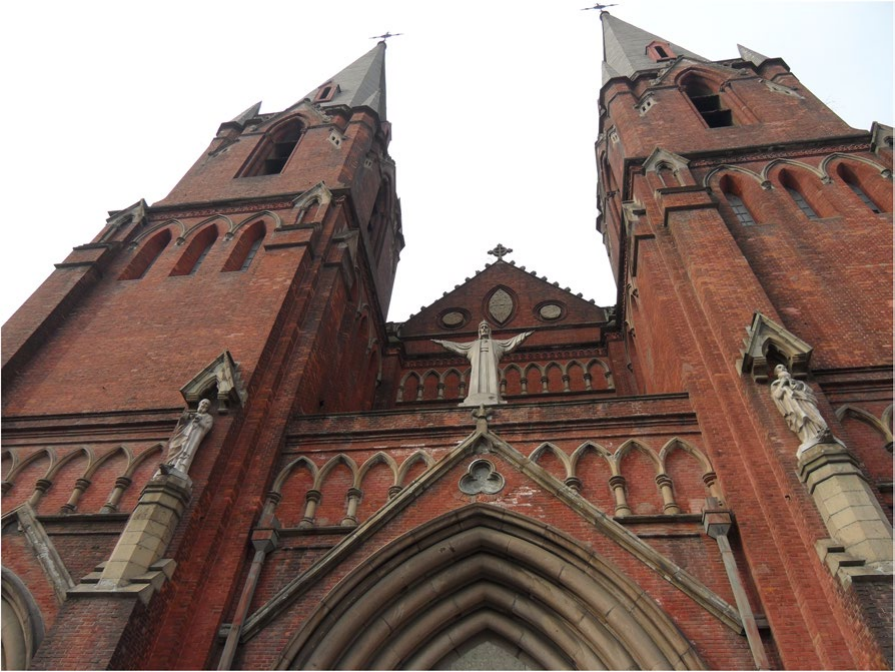
Stone elements of Xujiahui Cathedral (built between 1906 and 1910) in Shanghai before restoration in 2010. Besides granite, a greenish stone has been used, which might come from Ningbo (Source: Shibing Dai)

**Fig. 2:**
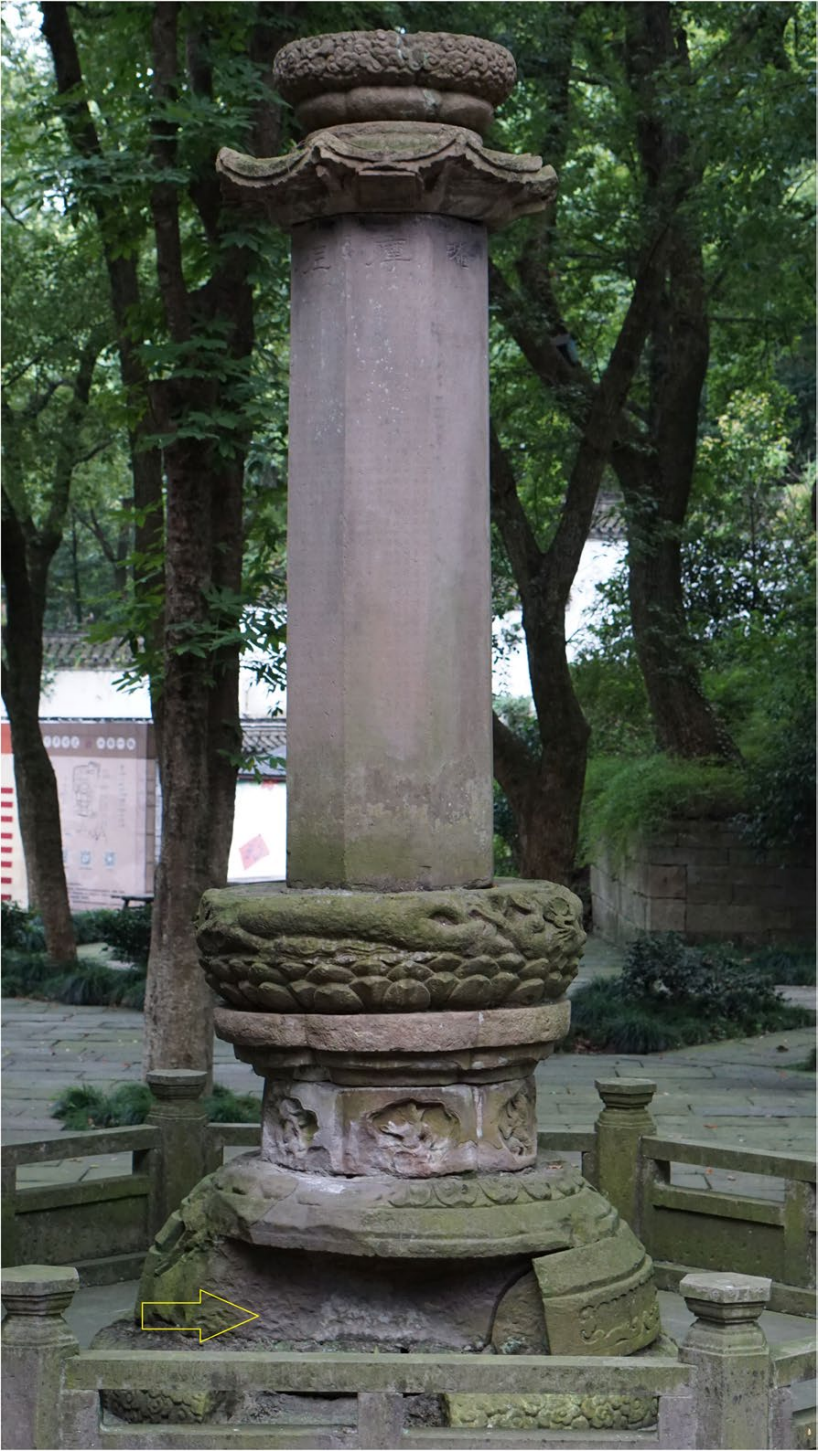
One of the two Buddhist stone pillars in front of Entrance of Baoguosi Temple in Ningbo. Two pillars might have been built in Tang Dynasty (618–907 CE). Missing stones need to be repaired/replaced using the same types of stone (Source: Shibing Dai)

However, the identification of the sources of natural stones plays also an important role for conservation and restoration of built heritage and monuments (Doehne and Price 2010). Due to natural and anthropic factors, some of stone elements of monuments and architectural heritage are either eroded or damaged. Those missing parts need to be restored for integrity and durability of built heritages (Figs. 1 and 2). In the context of authenticity and physico-chemical compatibility, stones of similar types shall be used for replacement or repairs especially to those monuments of high class (Jones 2006).

There are, however, not always documentation in written for sources of stones used as the heritage was built or restored. Therefore, mineralogical and chemical analyses can be explored to provide fundamental information about the characteristics of existing stones built in heritage and monuments. Even if there are some archives on the source of built natural stone, those statements need to be scientifically proven.

**Fig. 3:**
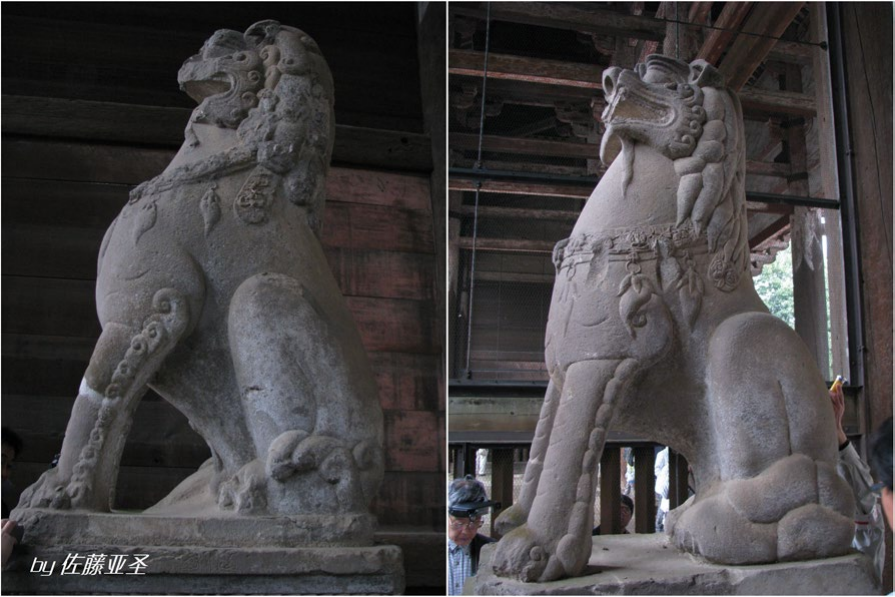
Stone Lions at the entrance of Todaiji Temple in Nara, Japan (Source: Satou Asei)

In order to characterise the stones in the City of Ningbo, where natural stones were quarried for over 20 centuries, a research project was launched before the Covid-19 pandemic. One of inspired reasons for the research project was that there was historical documents and publications showing the stone lions at the entrance of Todaiji Temple in Nara, Japan (Fig. 3), which is listed as UNESCO World Cultural Heritage, would have been made of Meiyuanshi Stone, which came from Ningbo (Oki et al. 2009). There is also confusion among historians and artists in Ningbo during architectural and archaeological surveys what is the genuine Meiyaunshi-stone.

**Fig. 4:**
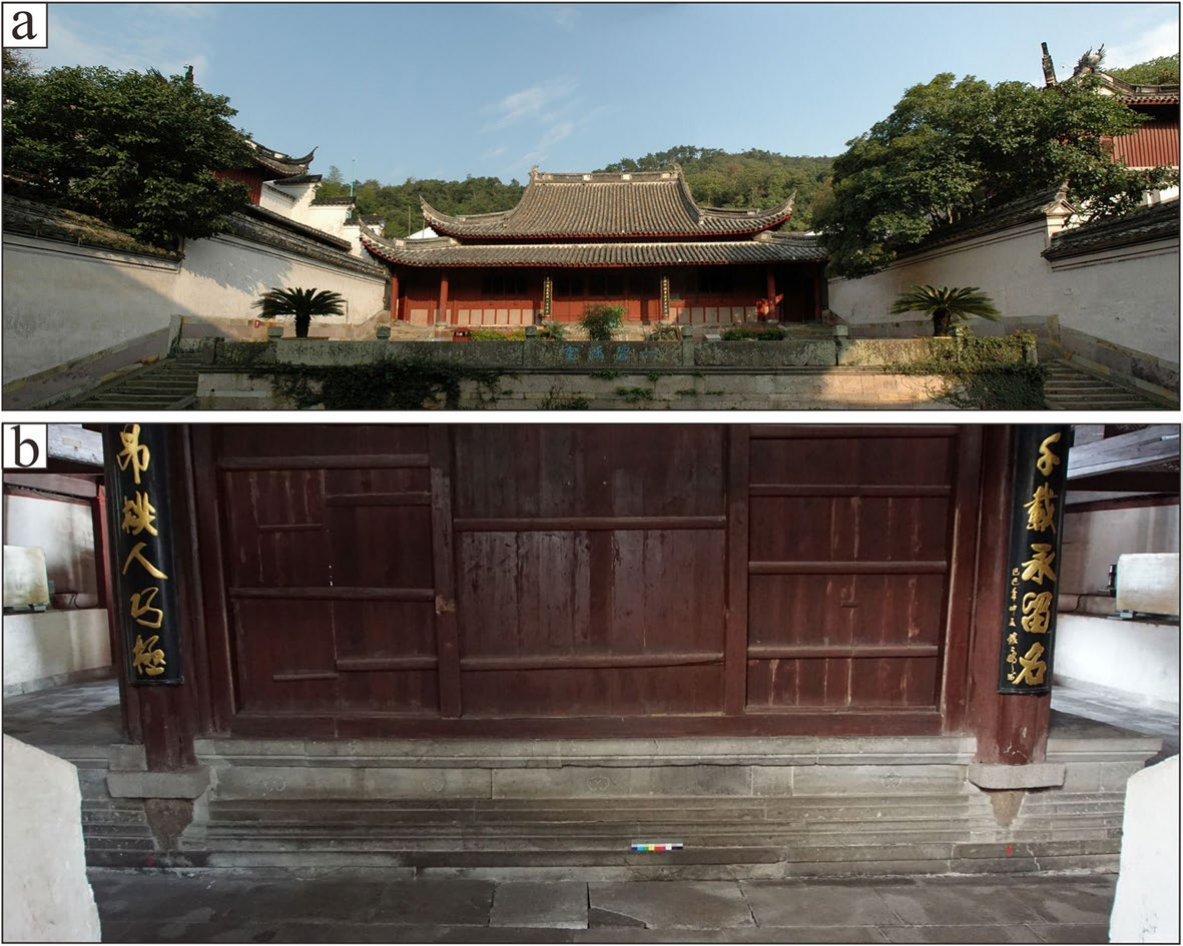
**a**: The southern façade of Baoguosi temple. **b**: The Sumeru platform within the main building of Baoguosi Temple (Source: Shibing Dai)

In the following text, the mineralogical and chemical data on three types of common stones from historic quarries, which are listed as historical sites, and on a sample form the Baoguosi Temple (Fig. 4) were reported. Although some of historic quarries are shut down, there are always possibilities to find materials which show clues of geological settings. There are also novelties in historical quarries, but geochemically the natural stones have certain similarities.

## Geological setting

**Fig. 5:**
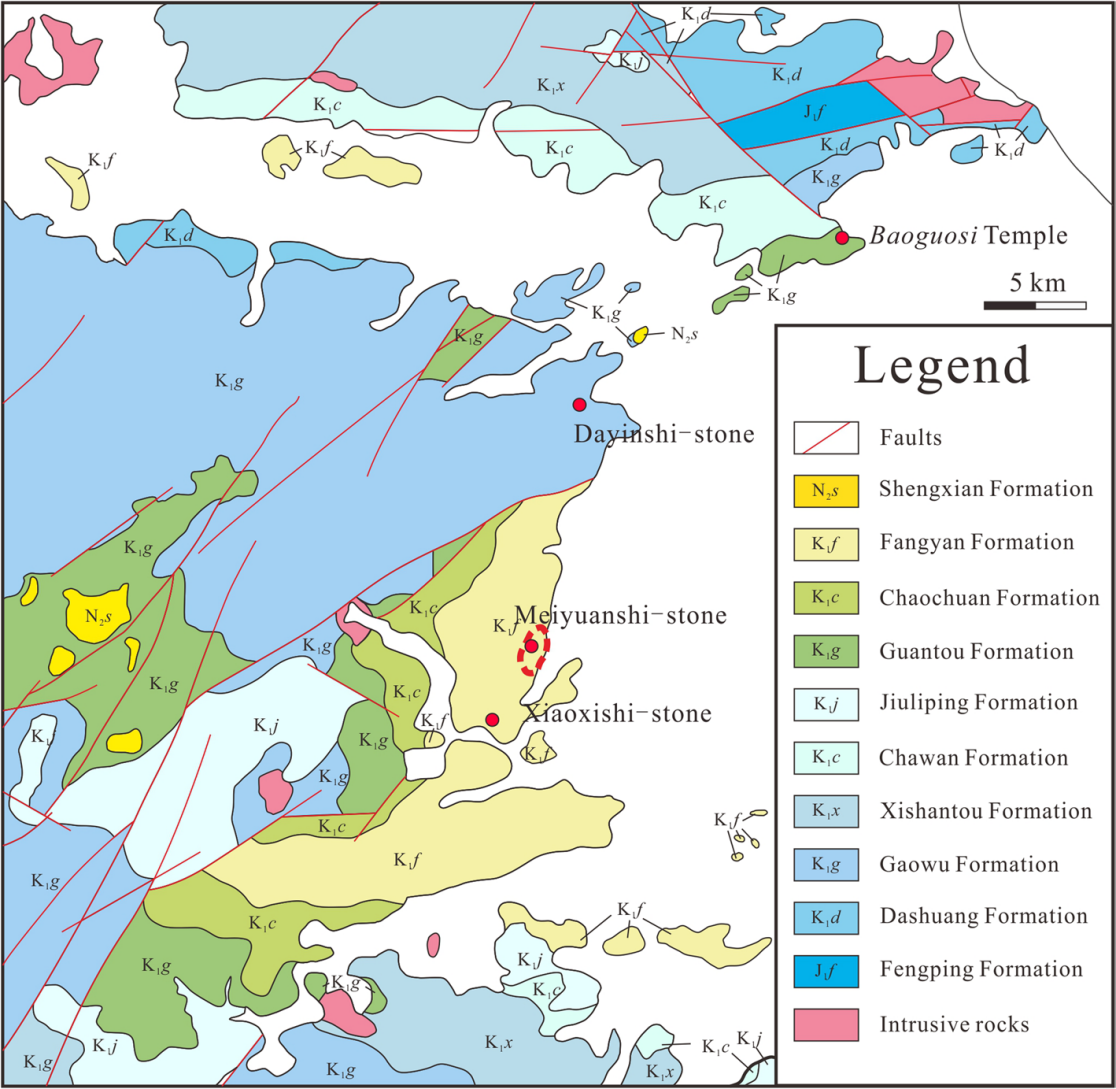
Simplified geological setting map of Ningbo and adjacent area. Location of the city of Ningbo indicating the geological epoch of the various formations and rocks. (Source: the authors, modified after BGMRZP 1989)

All quarries for the three famous stones of Ningbo are located at the Simingshan Mountain, westward of Ningbo City (Fig. 5). Tectonically, the Simingshan Mountain is situated in a Late Mesozoic volcanic relief (Cai and Yu 2001; Xu et al. 2018), which is associated with the extensional background (BGMRZP 1989, 344).

The volcanic activities in the Simingshan Mountain area were extremally active during Early Cretaceous. Volcanic rocks of the period widely distributed in adjacent area, accounting for about 90% of the exposed area of the base rock in this region. The dominant rocks are intermediate-acidic volcanic lava and pyroclastic rocks, while the subordinate rocks comprise acidic volcanic pyroclastic rocks, intermediate lava and a minimal amount of basic lava (Luo 2011). These lava and pyroclastic rocks in the Ningbo Basin can be divided into two parts according to stratigraphic classification, namely, the Moshishan Group of early Early Cretaceous and the Yongkang Group of late Early Cretaceous (Lapierre et al. 1997; Yu and Xu 1999). Isotopic dating, petrological, geochemical, and geomagnetic investigations have been performed for each group (Lapierre et al. 1997; Tao et al. 2000), suggesting that they are the products of two separated volcanic events (Cai and Yu 2001). Furthermore, Moshishan Group and Yongkang Group were dated approximately 135–121 Ma and 111–102 Ma, respectively, which implies the unconformity between them (Yu and Xu 1999).

## Material and methods

Stratigraphically, Dayinshi-stone was quarried from Gaowu Formation of Moshishan Group, whereas Meiyuanshi-stone and Xiaoxishi-stone were quarried from Fangyan Formation of Yongkang Group (Fig. 5). Gaowu Formation is dominated by rhyolitic welted tuff featured with high percentage of coarse crystal grains and a few interlayers of tuffaceous sandstone (BGMRZP 1989, 348; Luo 2011). Whereas Fangyan Formation is mainly consisted of sedimentary rocks and only a few interlayers of acidic tuff, the grain size of which get finer both northward and upward (BGMRZP 1989, 173–175).

The Baoguosi Temple is located in Lingshan Mountain, 13 kms northwest of Ningbo City (Fig. 5). Baoguosi Temple was originally built in 880 CE (Tang Dynasty, 618-907 CE). However, the buildings did not survive because of the slow development of Buddhism and unfavourable social conditions in this period. With the prosperity of Buddhism in Northern Song Dynasty (960-1127 CE), Baoguosi Temple was rebuilt and became one of the most valuable Buddhism Temple in China. The main building (Fig. 4a) was completed in 1013 CE after 6 years of construction. Over the last millennia, the monument was subjected to multiple expansion and restoration works (Guo 2003). According to oral documents, most stonework of the pavement in the building might have been replaced using local available volcanic rocks during this process, with an exception of the Sumeru platform (Fig. 4b). The platform was donated in 1102 CE and becomes the only remained stonework of Song Dynasty. Petrological classification and chemical features of the Sumeru platform have never been confirmed (SBCEC 2016), which might cause serious problems in case the monument demands replacement or conservation.

**Fig. 6:**
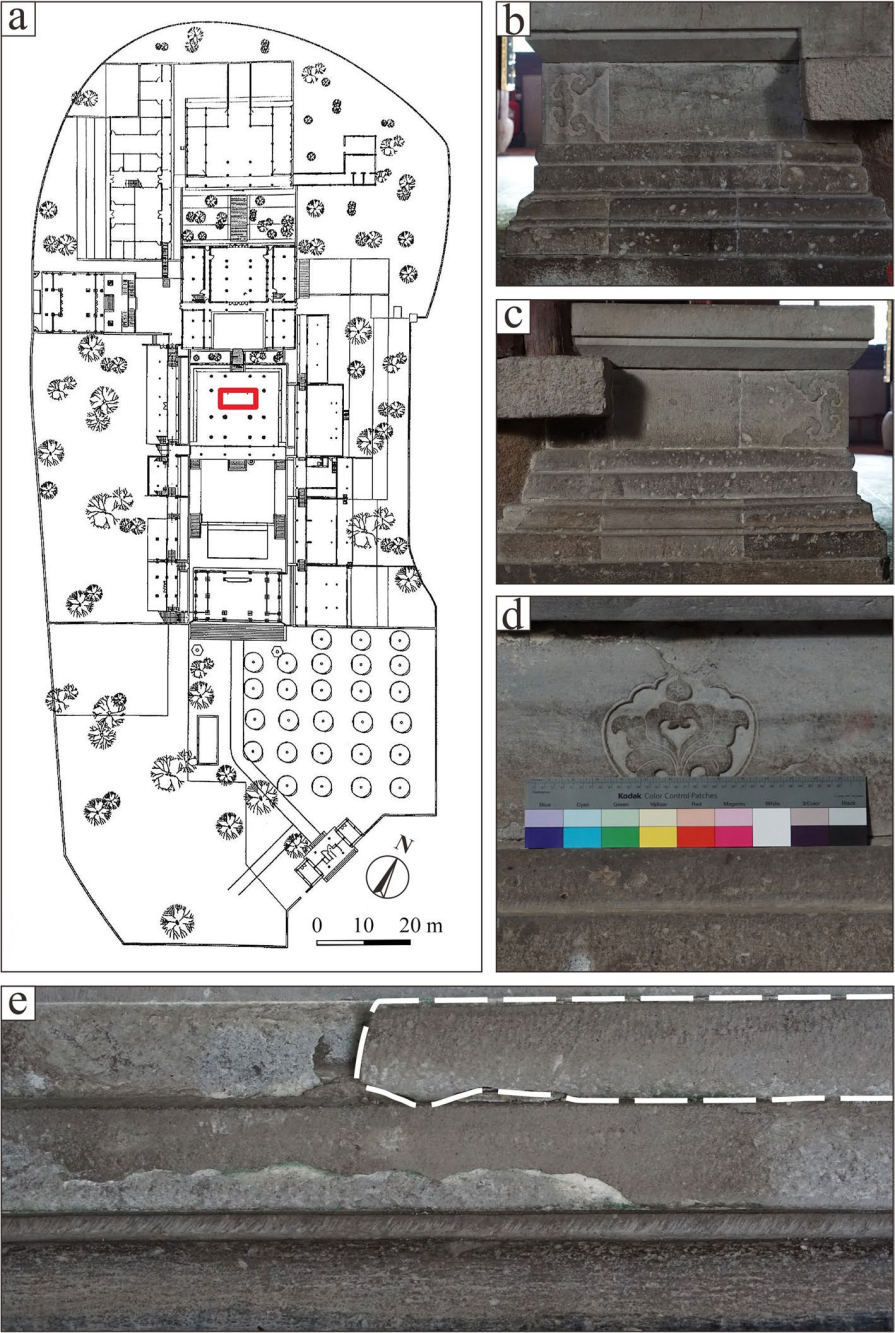
Sketch plane of Baoguosi Temple. **a**: The red rectangle indicates the location of the Sumeru platform. **b**, **c**, **d**: Details of the Sumeru platform. **e** (the height of picture is app 30 cm): The sampling position within the white broken line (Source: Guo 2003; Shibing Dai)

Detailed geochemical and petrographic investigations were carried out on each type of stones mentioned above, as well as sample retrieved from Baoguosi Temple (referred to as BGS) in Ningbo (Fig. 6). Petrographic and geochemical investigations are destructive, requiring rock samples to get thin sections for microscopic observation. Hence, for the samples from Baoguosi Temple, the research was addressed to those items which detached naturally from the monument. The heterogeneity of materials from monuments was not considered due to the limited quantity.

**Fig. 7:**
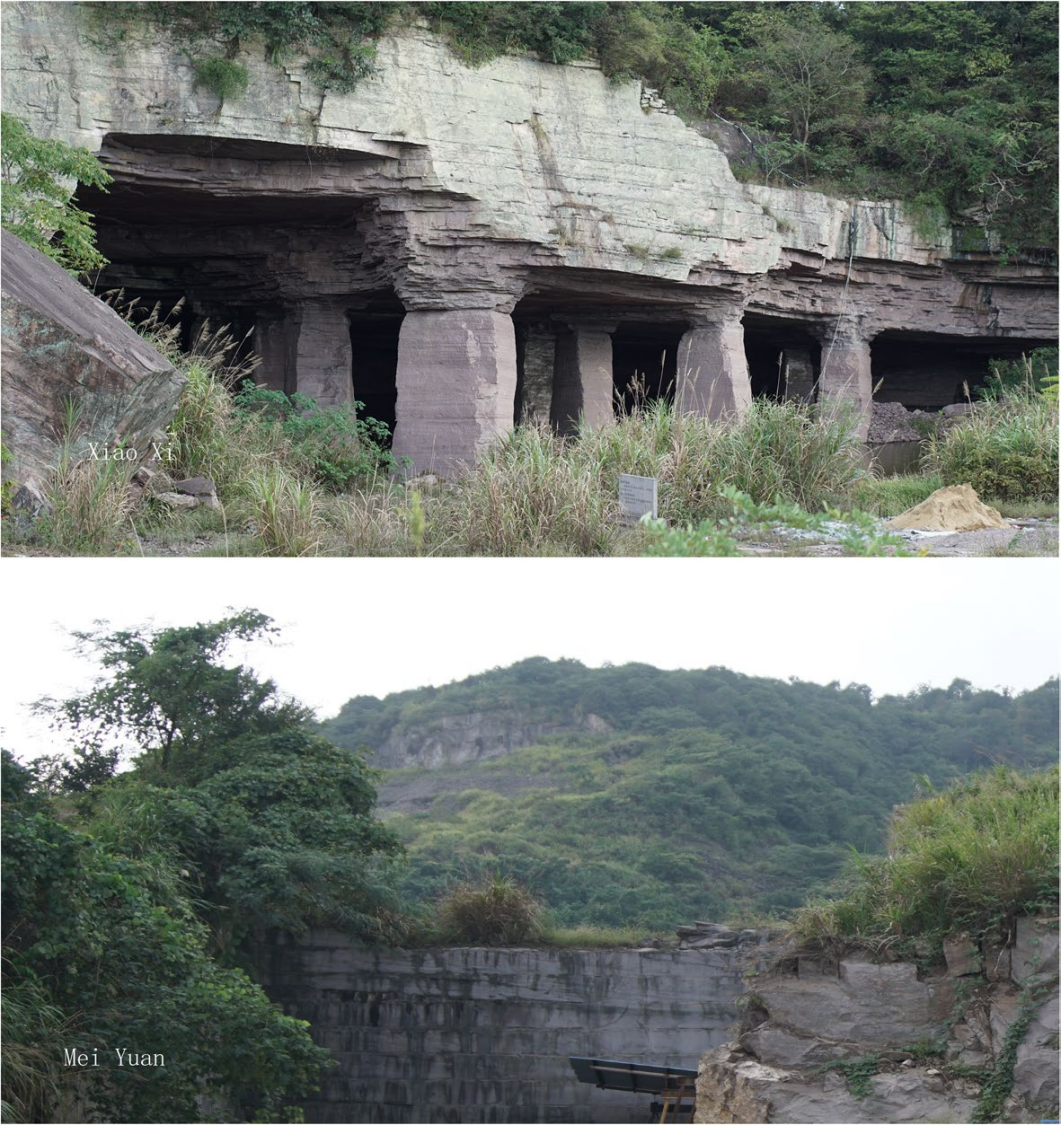
Historical quarries of Xiaoxishi Stone (upper photo) and Meiyuanshi Stone (lower photo), both are now listed as historical relics under protection (Source: Shibing Dai)

The thin section investigation was performed for all 11 samples of Meiyaunshi-stone (three samples), Xiaoxishi-stone (three samples), Dayinshi-stone (four samples) from quarries (Fig. 7), and an extra sample from Baoguosi Temple (Table 1). Among them, seven representative samples were selected for further analyses. The samples for geochemical composition determination were prepared using mixed powders of approximately 200 mesh. The major and trace element compositions were determined using Agilent Model 7900 Inductively Coupled Plasma Mass Spectrometer. Pervious work indicated that the analytical precision was better than 5% for major elements and 10% for trace elements (Rudnick et al. 2004). X-Ray diffraction (XRD) measurements of the samples carried out using SmartLab Rigaku X-ray diffractometer with Cu Kα radiation. The voltage and the current of the X-ray tube were 25 kV and 25 mA respectively. The profiles of all the samples were recorded in the angular range of 10–80° with a step size of 0.02° and a dwell time of 4s.

**Table 1:** List of analysed samples

Type	Sample Number	Description
Meiyuanshi-stone	M201809-1	Tuff, grey-purple to grey green, structureless
	M201809-2	
	M201809-2	
Xiaoxishi-stone	X201809-1	Tuff, light purple to purple, layered texture, show features of welted tuff
	X201809-2	
	X201607	
Dayinshi-stone	D201809-1	Tuff and sandstone, grey-green to reddish, layered texture
	D201809-2	
	D201809-3	
	D201809-4	
Baoguosi Temple	BGS	Tuff, light purple-grey, structureless

## Results

### Petrographic characteristics

**Fig. 8:**
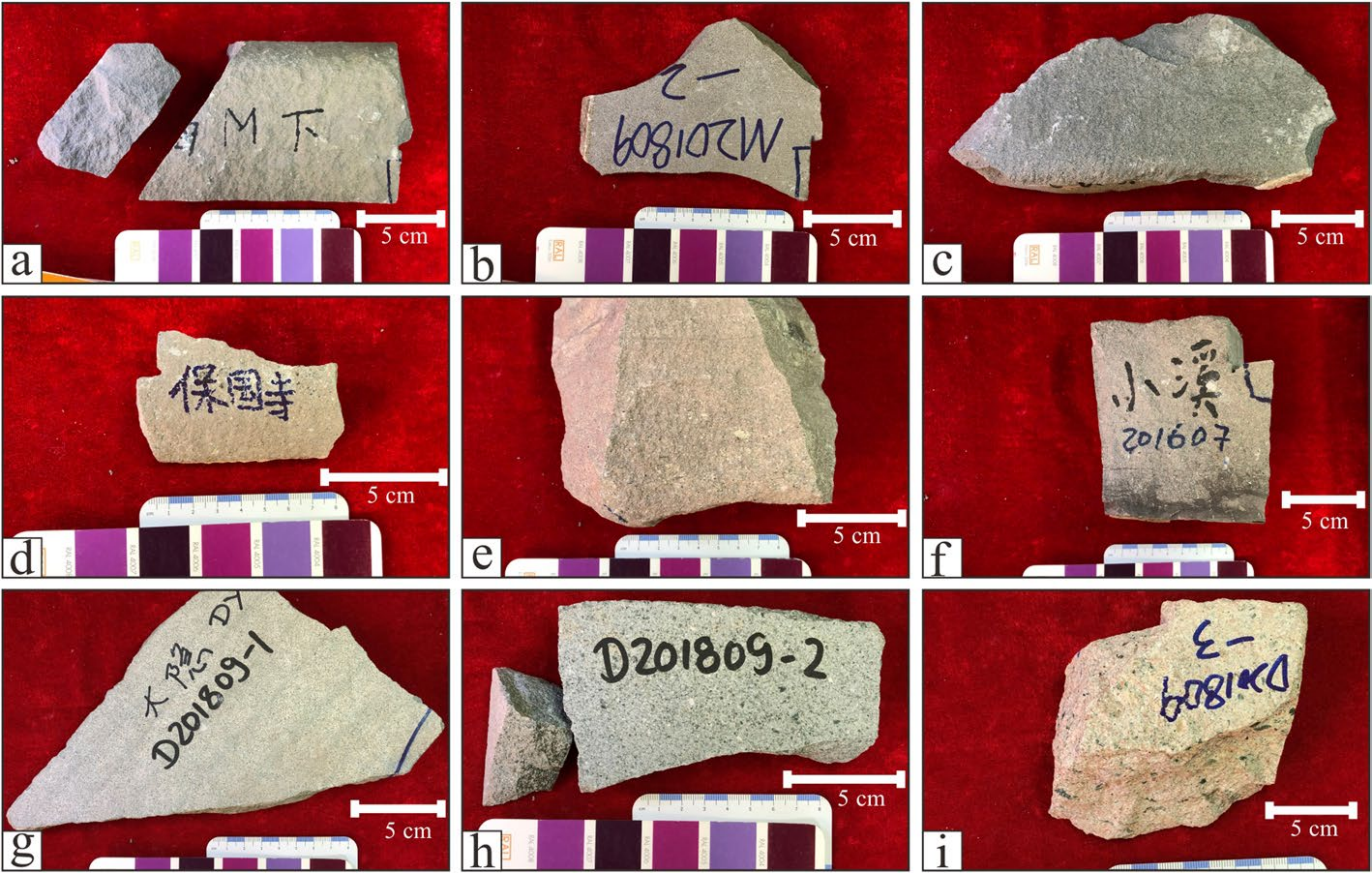
Photographical documentation of samples for mineralogical and chemical analyses: (**a**) M201809-1; (**b**) M201809-2; (**c**) M201809-3; (**d**) BGS; (**e**) X201809-1; (**f**) X201607; (**g**) D201809-1; (**h**) D201809-2; (**i**) D201809-3 (Source: Shibing Dai)

All three types of stones are volcanic clastic rocks from petrographic point of view (Fig. 8). Although the percentages of fragments differ, they all consist of crystal and rock clasts of intermediate-felsic volcanic rock (dacite or andesite).

#### Meiyuanshi-stone

**Fig. 9:**
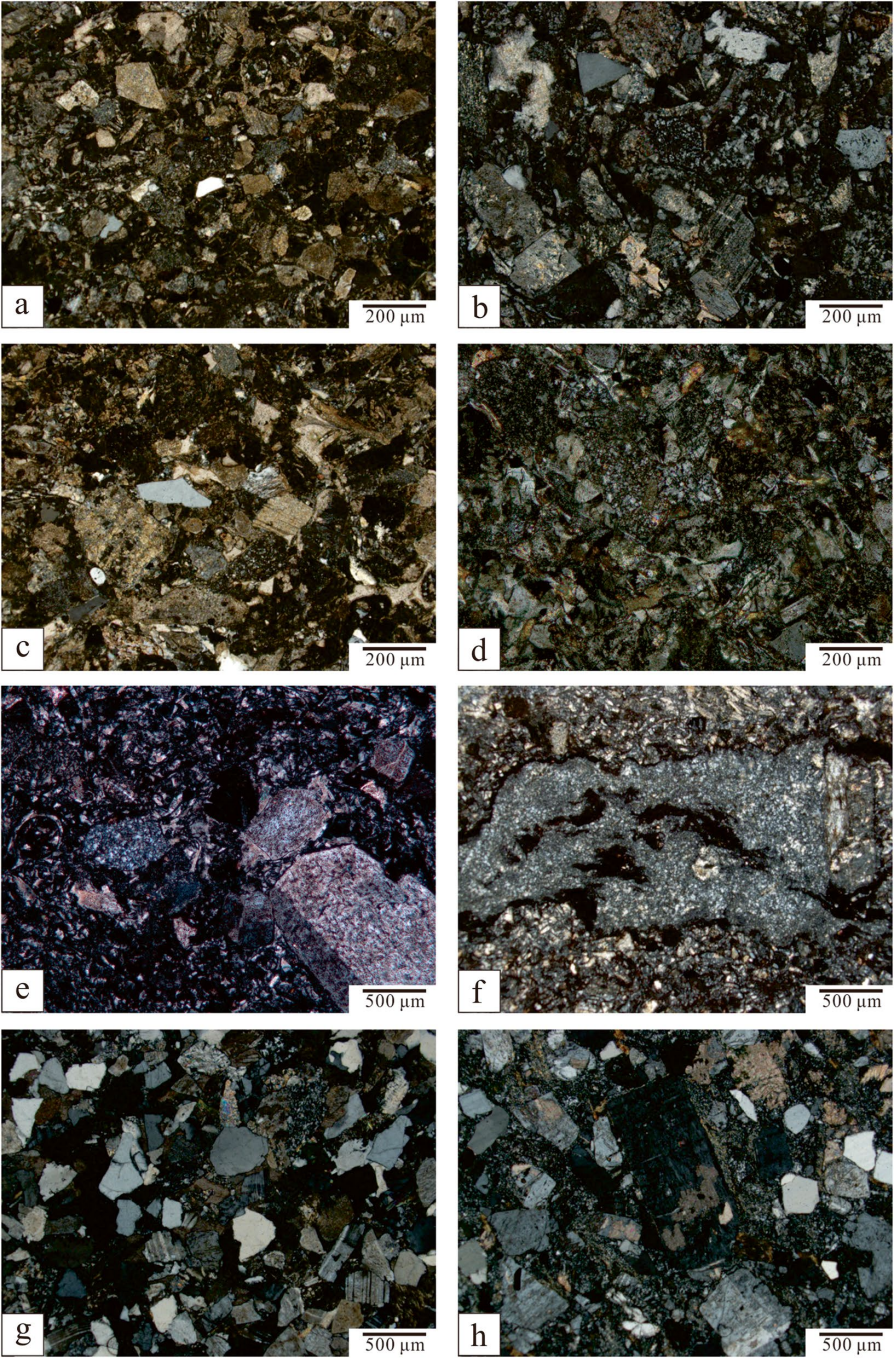
Microphotographs of thin sections by polarised light microscope (all images were taken with crossed Nicols): (**a**) M201809-1; (**b**) M201809-2; (**c**) M201809-3; (**d**) BGS; (**e**) X201809-1; (**f**) X201809-2; (**g**) D201809-1; (**h**) D201809-2 (Source: the authors)

The outcrops have no banding or inclusions of other materials. The hand specimens are structureless and massive. The constituent materials show no orientation under polarised microscope and are mainly tuff-grade volcanic clasts (with grain sizes varied from 0.2 to 0.5 mm, concentratedly distributed in 0.2 - 0.3 mm) which can be identified as crystal and rock clasts. They are clast-supported and cemented by ash matrix (Fig. 9a, b, c). However, the colour may differ from light purple to greyish green, which might relate to the iron composition.

#### Xiaoxishi-stone

Petrographic study of typical thin section of samples shows layered texture, which is framed by two different grain sizes of layers. The grain size in the coarser layers varied from 0.2–0.8 mm (up to 2.0 mm) with 0.03–0.05 mm cement, while the finer layers have the grain sizes of 0.1–0.3 mm (Fig. 9e). The mineral contents in the two layers, however, are quite similar. Some specimens show features of incipiently welted tuff (Fig. 9f).

#### Dayinshi-stone

The total four samples of Dayinshi-stone from the single quarry can be classified into tuff (three samples) and sandstone (one sample). Compared to the tuff samples (D201809-2; Fig. 8h, Fig. 9h), the sandstone sample (D201809-1; Fig. 8g, Fig. 9g) has more terrestrial debris (70%). Although poorly sorted, the terrestrial debris have better roundness than the volcanic ones. The clasts are cemented mainly by carbonate minerals rather than ash in pyroclastic rocks.

As observed from a single thin section, the Dayinshi-stone contains multiple layers, with the thinnest thickness of a few millimetres. These layers vary significantly in mineral contents and grain sizes. Dayinshi-stones are poorly sorted and low in volcanic clast contents, with fragments size varies from below 0.01 mm to 8 mm. Correspondently, the rock fragments have multiple types, both considerable amount of andesite and dacite fragments were identified. Over all, the most pronounced feature of Dayinshi-stone would be presentation of polymictic breccia clasts (Fig. 9i).

#### Sample of Baoguosi Temple (BGS)

The sample retrieved from the Sumeru platform in Baoguosi Temple shares much similarities with Meiyuanshi-stone. The hand specimen is light purple-gray, structureless, and emplaced in massive. Micro-petrographically, it is dominated with crystal and rock clasts of 0.2–0.8 mm (80%), as well as a small portion of pyroclastic ash (< 0.05 mm; approximately 20%) (Fig. 9d). The crystal clasts, distributed within the grain size of 0.10- 0.25 mm, are mainly composed of feldspars with few quartzes, while the rock clasts are andesitic, dacitic or trachytic.

### Geochemical compositions

**Fig. 10:**
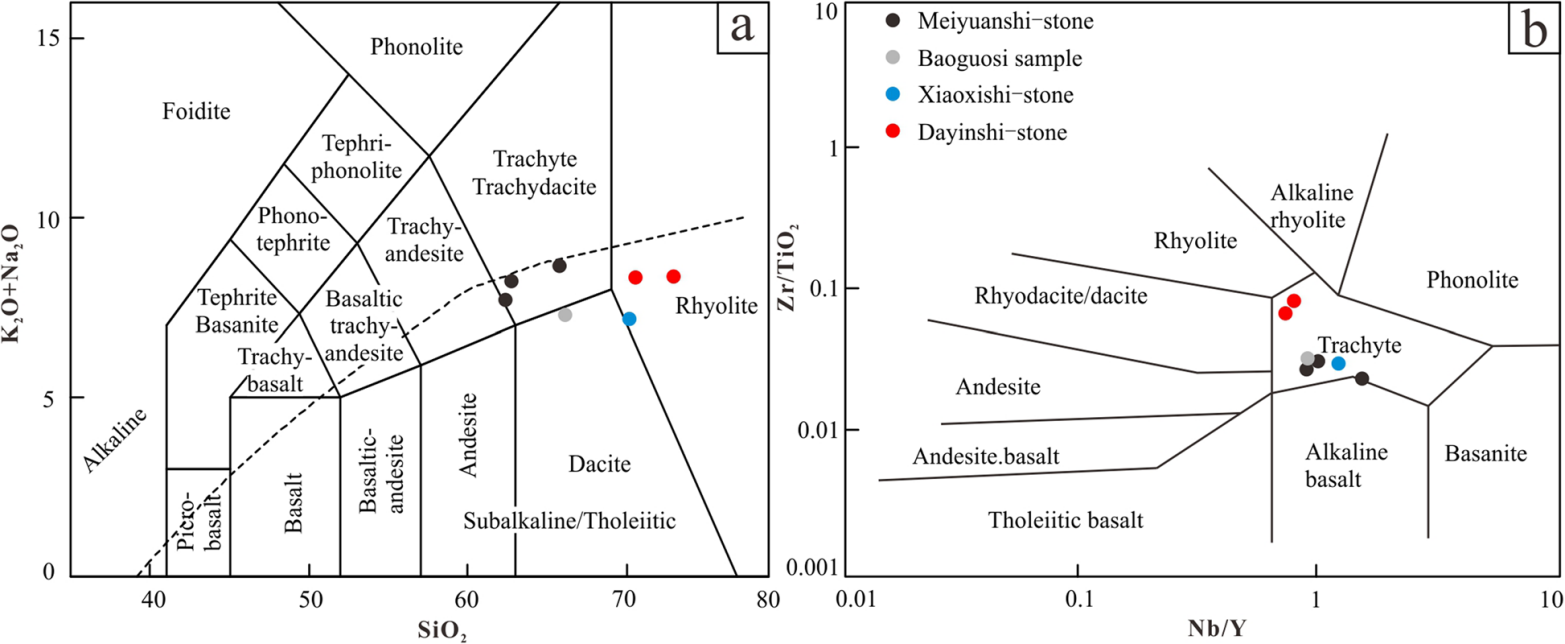
Diagram of selected samples. **a**: Total alkali-SiO_2_ (TAS) diagram (Source: the authors, after Le Bas et al. 1986). **b**: Zr/TiO_2_-Nb/Y diagram (Source: the authors, after Winchester and Floyd 1977)

Seven samples of the three kinds of stones were analysed for both major (Table 2) and trace elements (Table 3). The Meiyuanshi-stone samples have SiO_2_ of 64.7- 67.3 *wt.*%, Al_2_O_3_ of 15.1–15.4 *wt.*%, and total alkalis content of 7.9–8.8 *wt.*%. The contents of MgO, TFe_2_O_3_ and Mg# values of these rocks are 1.8–2.5 *wt.*%, 4.2–5.7 *wt.*%, and 46–49, respectively. Total alkali-SiO_2_ (TAS) diagram shows that all Meiyuanshi-stone samples have the composition equivalent to trachyte or trachydacite (Fig. 10a). In contrast, the Dayinshi-stone samples, which have relatively high SiO_2_ (71.8–74.2 *wt.*%), similar total alkalis (both 8.4 *wt.*%) but lower Mg# values (24–36), were plotted in the rhyolite field (Fig. 10a). While the contents of MgO (0.27–0.75 *wt.* %), TFe_2_O_3_ (1.73–2.61 *wt.* %), and TiO_2_ (0.20–0.34 *wt.* %) are lower than those of Meiyuanshi-stone samples, but with similar CaO content (1.47–1.73 *wt.* %). Plotted in rhyolite area, the Xiaoxishi-stone sample shows much similarities with Dayinshi-stone in major elements, such as SiO_2_ (72.2 *wt.*%), total alkalis (7.3 *wt.*%), MgO (0.81 *wt.*%), TFe_2_O_3_ (3.06 *wt.* %), and TiO_2_ (0.40 *wt.* %) contents. Although the Baoguosi sample falls into the dacite area in TAS diagram, it shares consistent SiO_2_ (68.4 *wt.*%), total alkalis (7.5 *wt.*%), MgO (1.3 *wt.* %), TFe_2_O_3_ (3.6 *wt.* %), and TiO_2_ (0.53 *wt.* %) contents with Meiyuanshi-stone.

**Table 2:** Major elements content of selected samples

Elements	M201809-1 (*wt.* %)	M201809-2 (*wt.* %)	M201809-3 (*wt.* %)	BGS (*wt.* %)	X201809-1 (*wt.* %)	D201809-2 (*wt.* %)	D201809-3 (*wt.* %)
SiO_2_	65.80	62.74	62.33	66.09	70.22	70.58	73.02
TiO_2_	0.55	0.62	0.71	0.51	0.39	0.33	0.20
Al_2_O_3_	14.82	14.86	14.56	14.71	12.95	14.03	12.83
Fe_2_O_3_	4.08	4.60	5.48	3.44	2.98	2.57	1.7
MnO	0.11	0.13	0.17	0.09	0.12	0.16	0.08
MgO	1.75	2.22	2.36	1.27	0.79	0.74	0.27
CaO	1.62	2.96	2.59	3.18	2.23	1.45	1.7
Na_2_O	5.83	5.46	3.75	4.72	2.95	4.47	5.03
K_2_O	2.77	2.70	3.89	2.56	4.18	3.82	3.28
P_2_O_5_	0.15	0.17	0.19	0.11	0.10	0.09	0.04
LOI	2.22	3.51	3.69	3.16	2.74	1.67	1.58
Total	99.71	99.97	99.71	99.85	99.64	99.85	99.74

**Table 3:** Trace elements content of selected samples

Elements	M201809-1 (ppm)	M201809-2 (ppm)	M201809-3 (ppm)	BGS (ppm)	X201809-1 (ppm)	D201809-2 (ppm)	D201809-3 (ppm)
Li	28.44	38.27	46.4	27.08	20.4	35.22	18.62
Be	1.582	1.474	1.862	1.906	1.938	1.911	1.88
Sc	5.476	6.417	3.999	5.912	2.39	3.605	3.61
V	69.82	97.98	116.7	70.72	16.69	35.03	28.5
Cr	28.73	33.57	34.35	18.4	15.52	6.649	11.41
Co	10.84	12.35	14.15	6.737	1.784	3.607	3.461
Ni	13.8	14.81	14.94	8.522	3.5	3.353	3.334
Cu	5.708	3.644	9.994	18.55	4.455	5.209	4.624
Zn	60.47	80.19	129	57.1	21.45	61.13	49.21
Ga	16.05	17.04	16.4	15.68	12.05	15.27	16.07
Rb	15.61	12.88	12.3	22.65	61.69	75.2	70.56
Sr	161.8	111.6	75.46	377.1	206.1	242.7	216.3
Y	12.78	12.62	7.118	13.91	10.49	15.38	15.46
Zr	174.2	173.9	171.9	170.3	119	220.1	164.2
Nb	12.8	11.3	10.93	12.58	12.77	11.25	12.25
Cs	0.8386	1.063	1.917	4.395	1.351	8.734	3.455
Ba	818.4	438.8	329.2	557.6	650.8	1245	985.2
La	17.22	21.48	13.43	26.74	23.56	48.8	43.85
Ce	36.01	42.93	34.69	52.11	42.45	81.87	76.28
Pr	4.333	5.147	3.708	6.103	4.923	9.3224	8.548
Nd	16.16	19.44	14.17	21.64	16.76	30.97	28.01
Sm	3.02	3.531	2.725	3.995	2.955	4.909	4.557
Eu	0.7616	0.9875	0.7319	1.026	0.6932	1.099	1.058
Gd	2.748	3.169	2.289	3.299	2.382	4.057	3.883
Tb	0.4158	0.4398	0.3465	0.4636	0.3434	0.5533	0.5093
Dy	2.454	2.607	1.975	2.66	1.875	2.929	2.922
Ho	0.5209	0.5138	0.4054	0.5265	0.3814	0.5735	0.5905
Er	1.534	1.533	1.234	1.55	1.137	1.605	1.763
Tm	0.2356	0.2301	0.1831	0.2359	0.1768	0.2342	0.2677
Yb	1.534	1.558	1.195	1.545	1.19	1.565	1.808
Lu	0.2278	0.2316	0.1844	0.232	0.1816	0.2442	0.2802
Hf	9.929	9.888	9.738	9.665	7.358	11.97	9.536
Ta	0.9123	0.813	0.7208	0.8727	0.9396	0.6726	0.9108
Tl	0.549	0.5827	0.5925	0.4957	0.562	0.7394	0.6751
Pb	11.36	9.971	8.302	33.45	11.43	27.08	22.24
Th	8.781	7.286	2.472	10.22	11.65	13.71	15.93
U	2.942	2.268	2.126	3.107	5.251	2.245	3.513

Major elements contents in rocks would suffer noticeable alteration from weathering, while trace elements show insensitivity against it. Hence, as a complementary classification by the TAS diagram, the diagram of Zr/TiO_2_-Nb/Y (Fig. 10b) was employed, where all samples fall into trachyte area.

**Fig. 11:**
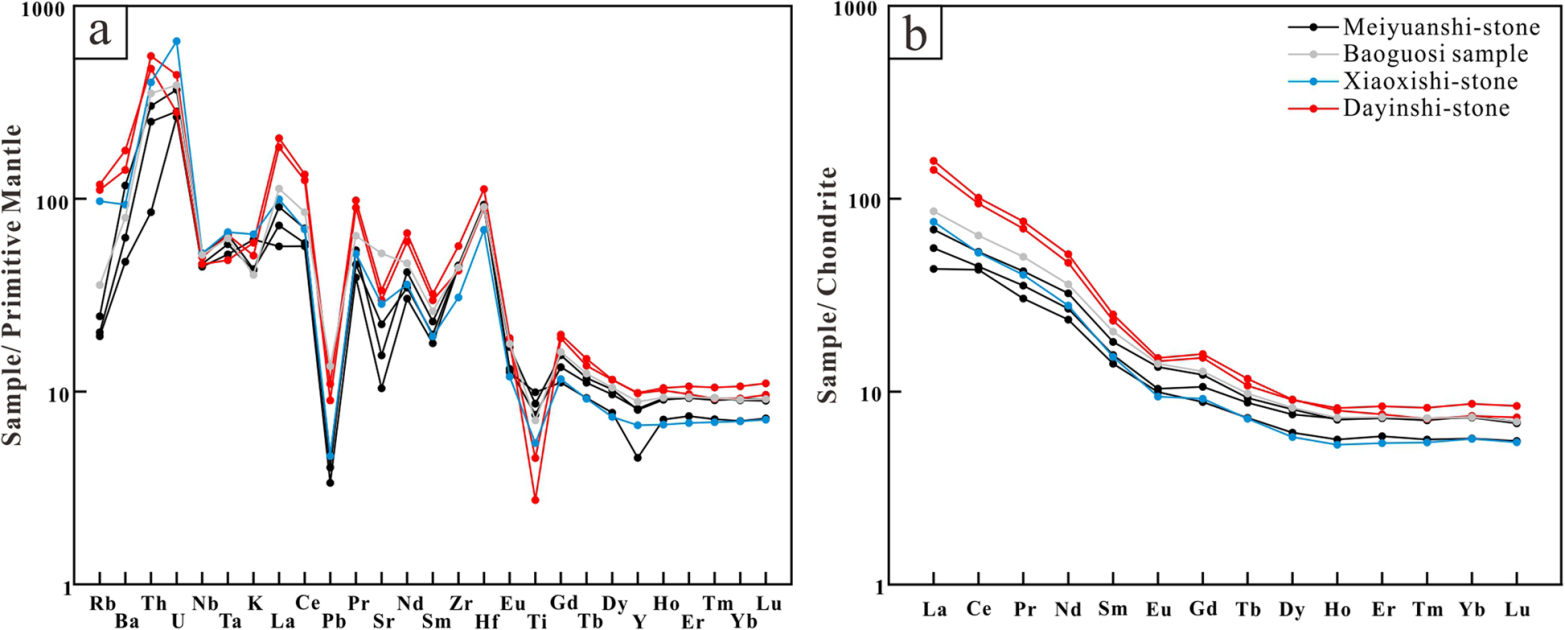
**a**: Primitive mantle-normalised spider-grams for trace elements (Source: the authors, after Sun and McDonough 1989). **b**: Chondrite-normalised rare earth elements diagram (Source: the authors, after Boynton 1984)

In primitive mantle-normalised spider-grams (Fig. 11a), the seven curves can be categorised as two evidently separated groups. (1) Firstly, the Dayinshi-stone samples show equivalent trace element patterns with enrichment of Ba, Rb, Pb, Zr, and Hf, but depletion of Nb, Ta, Pb, and Sr, comparable to average continental arc andesites (Kelemen et al. 2003). Such patterns are common for the continental crust that originated from the geochemical evolution of an arc-derived magma chamber (Hawkesworth and Kemp 2005; Rudnick and Gao 2003). (2) Meiyuanshi-stone as well as the Baoguosi sample have similar trace element distribution patterns with the Dayinshi-stone samples, except for the depletion of Rb and Sr. Xiaoxishi-stone is almost identical with Meiyuanshi-stone in trace elements patterns, except the less dramatic depletion of Rb.

As demonstrated in the chondrite-normalised rare earth elements diagram (Fig. 11b), Dayinshi-stone samples show consistent patterns of enrichment of light rare earth elements (LREE) with (La/Yb)_N_ ratios of 17.40–22.37 and pronounced negative Eu anomalies (δEu = 0.75–0.77), while all the other samples exhibit similar patterns with less enrichment of LREE ((La/Yb)_N_ = 8.05–14.20) and slightly less significant negative Eu anomalies (δEu = 0.80–0.90).

### X-Ray diffraction results

**Fig. 12:**
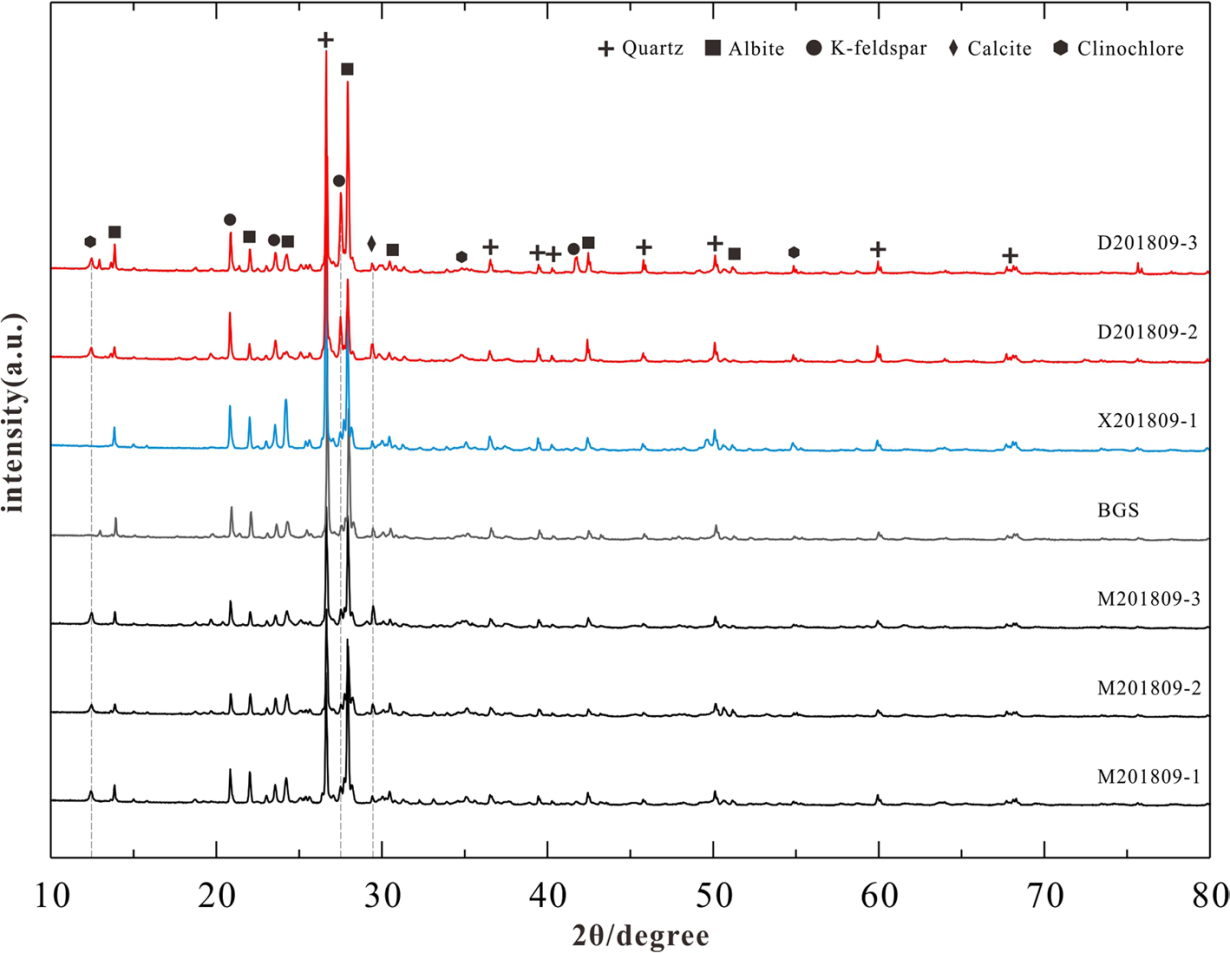
X-ray diffraction patterns of selected samples (Source: the authors)

XRD analysis suggested that all seven samples mainly consist of quartz, albite, K-feldspar, and clinochlore. The patterns of the Meiyuanshi-stone and the Baoguosi specimen are consistent except for slight differences on calcite content. Whereas Xiaoshishi-stone have similar specificities with Meiyuanshi-stone, but with more profound peak of albite. As for Dayinshi-stone, it is characterised with the considerable amount of K-feldspar (Fig. 12; Table. 4).

**Table 4:** Estimated mineral contents of selected samples by X-ray diffraction

Samples	Mineral Contents(*wt*.%)				
	Quartz	Albite	K-feldspar	Calcite	Clinochlore
M201809-1	22	57	20	minor	1
M201809-2	23	53	23	minor	1
M201809-3	24	59	14	2	1
BGS	28	54	18	minor	minor
X201809-1	31	47	22	minor	minor
D201809-2	42	18	38	minor	2
D201809-3	32	35	29	2	2

## Discussion

### Meiyuanshi-stone and Baoguosi sample

In the first place, the sample retrieved from the Sumeru platform in Baoguosi Temple (BGS) shares much similarities with Meiyuanshi-stone, both petrographically and geochemically. The hand specimens are light purple-gray and structureless. Micro-petrographically, it is dominated with crystal and rock clasts of 0.2- 0.8 mm (80%). The rock clasts are andesitic, dacitic or trachytic. Regarding the geochemical composition, the BGS sample is identical to the Meiyuanshi-stone in both major (Fig. 10) and trace elements contents (Fig. 11). Similarly, XRD patterns show consistence between them (Fig. 12). The absence of clinochlore peak in BGS sample might be a consequence of weathering of the stone, since clinochlore is not stable under normal pressure and temperature (Critelli et al. 2014).

The bureau of geology and mineral resources of Zhejiang Province (1989) referred Fangyan Formation as a set of terrigenous clastic rock like sandstone, siltstone, and conglomerate rocks. Former researchers also documented Meiyuanshi-stone as tuffaceous sandstone (Yan 1995; Jin 2010). The major references might be its emplacement in massive structure and evenness in the grain size (well-sorted). However, based on detailed petrological investigations in this paper as well as previous studies of Oki et al. (2009; 2010), it is more logical and rational to classify Meiyuanshi-stone as tuff due to its dominant pyroclastic composition (90%; Sun et al. 2001).

**Fig. 13:**
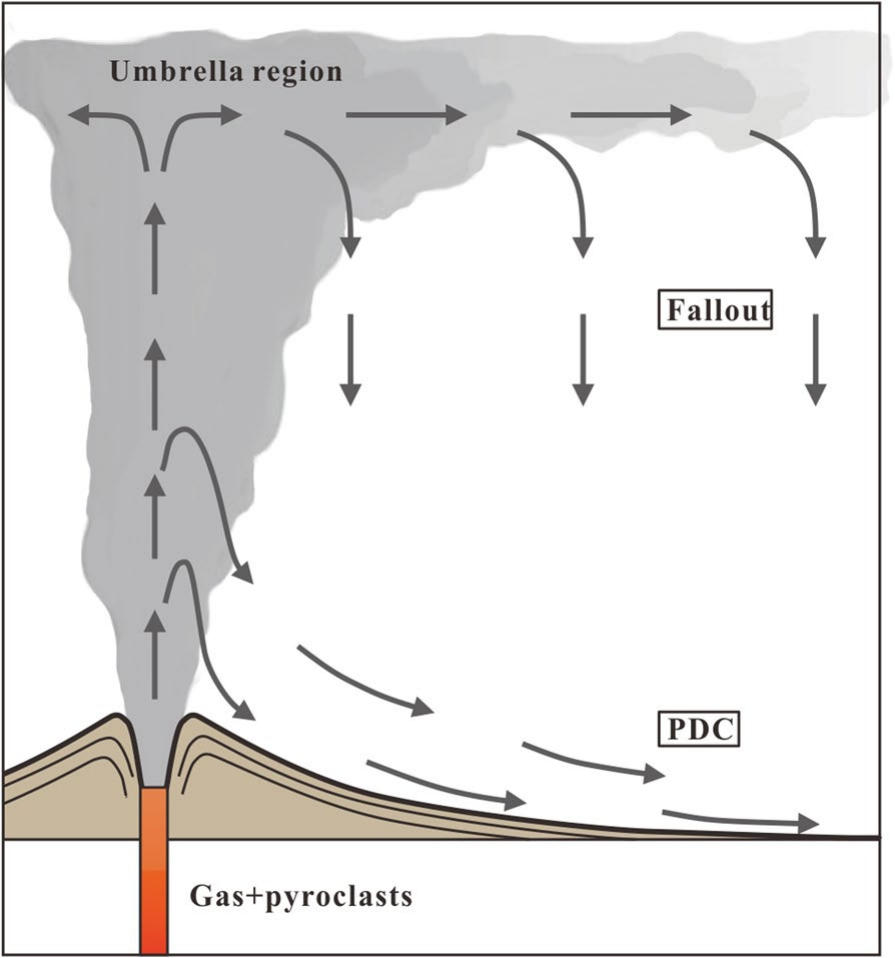
General scheme of fallout and pyroclastic density flow (PDC) processes in eruptions (Source: the authors, modified after Dowey and William 2022)

As mentioned above, besides Meiyuanshi-stone, Xiaoxishi-stone and some of Dayinshi-stone are also tuffs. Compared to other more common heritage building materials like marble, sandstone or granite, tuffs are usually considered more sensitive to weathering (Pötzl et al. 2022). The reasons often trace back to their dramatically changed depositional environments that cause a strong mineralogical and fabric heterogeneity (Acocella 2021; Fig. 13). Tuff is a kind of pyroclastic rocks that can be considered as sedimentary rocks as well as igneous rocks, for the materials come from the explosive volcanic eruption whereas the way they formed is by deposit (Peterson 1985). Tuffs could be results of either plume (fallout) or pyroclastic density current (mixtures of pyroclastic particles and gas that move across the landscape under the effect of gravity) activity (Dowey and Williams 2022).

### Relation between Meiyuanshi-stone and Xiaoxishi-stone

Should all the three kinds of natural stone materials be tuff, the physical properties of them can vary significantly according to the different lithologic categories and diverse depositional environments. In this case, Dayinshi-stone and Xiaoxishi-stone show heterogeneity like typical tuffs, while Meiyuanshi-stone doesn’t. Based on the geological investigation conducted by the Bureau of Geology and Mineral Resources of Zhejiang Province (1996, 136–174), Meiyuanshi-stone and Xiaoxishi-stone both belong to Fangyan formation. Furthermore, the two sampling sites of them are only approximately five kilometres away from each other (Fig. 5). The reason why they are so different may lay in the mechanism and behaviours of plume pyroclastic density currents during the eruption.

Many pyroclastic deposits are associated with extremely fine-grained deposits on top (at least 70% of the clasts are finer than 0.25 mm; Sparks 1976). These characteristics imply a fallout origin, but their intimate relation with the PDC deposits indicates that the two kinds of sedimentary mechanism can emplace during single volcanic eruption (Fig. 13; Acocella 2021). Until recently, depositional models of different pyroclastic density currents were developed by field observations, laboratory experiments, and numerical simulations (Sulpizio et al. 2010; Dellino et al. 2010; Dowey and Williams 2022). It has been recognised that the bigger clasts would go further before they fall and deposit because of higher kinetic energy (Sulpizio et al. 2014).

The similar rock and crystal clasts of the Meiyuanshi-stone and Xiaoxishi-stone confirms that they are formed from the same juvenile magma. Most trace-element concentrations change in a regular and predictable manner with progress of magma evolution (Wager and Mitchell 1951; Nockolds and Allen 1954). During the fractionation processes of magma generation and evolution, trace elements would enter solid or melt phase according to their compatibility. Trace elements are highly sensitive to partial melting and fractional crystallisation. Igneous rocks sharing similar features in trace elements usually derived from identical magma source and endured similar evolution process. The similar trace elements contents and distributions (Fig. 11) further imply the affinity between Meiyuanshi-stone and Xiaoxishi-stone. To sum up, Meiyuanshi-stone and Xiaoxishi-stone are likely formed from pyroclastic fallout and flow (generated during single eruption), respectively.

Based on aforementioned results, the stone utilised for the Pillars (Fig. 2) has been identified very likely as Meiyuanshi-stone.

### Dayinshi-stone

The significant heterogeneity of Dayinshi-stone often leads to ambiguous characterisation. Bureau of geology and mineral resources of Zhejiang Province defined Gaowu Formation, the unit where Dayinshi-stone were quarried, as rhyolitic welted tuffs featured with high percentage and coarse crystal pyroclastic, as well as few interlayers of tuffaceous sandstone. The description is compliant with our on-site investigation. By our investigation, 3 of total 4 samples (D201809-2, D201809-3, D201809-4) were identified as tuff while D201809-1 as sandstone, which may relate to the complicated sedimentary environment of the time.

For major elements, Dayinshi-stone and Xiaoxishi-stone show similar contents because of the evolution pattern of igneous rocks. Similarly, Rb/Sr ratios of Dayinshi-stone samples ranging from 0.30 to 0.33, whereas the ratio for Xiaoxishi-stone is 0.31. Due to the difference in the compatibility, Rb and Sr is highly sensitive to the process of magmatic evolution and generally shows a gradually increasing trend from basaltic to felsic rocks (Dasch 1969). Hence, the consistent major elements contents and Rb/Sr ratios of Dayinshi-stone and Xiaoxishi-stone indicates the same evolution stage instead of identical magma source.

### Sources of the stone from Xujiahui Cathedral Shanghai and other hisotirc buildings

**Fig. 14:**
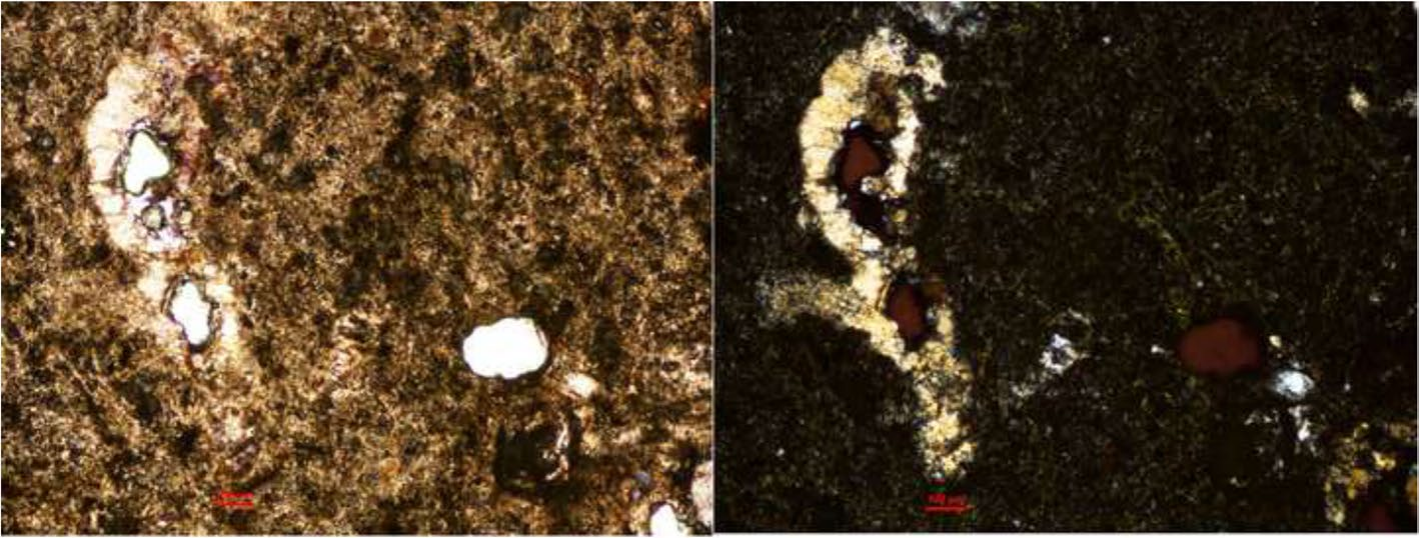
Microphotographs of a thin section taken from the heavily deteriorated greenish/grey stone from Xujiahui Cathedral Shanghai under polarised light microscope. Left: taken with singular. Right: with crossed Nicols (Source: the authors)

Based on the preliminary study, the greenish natural stone from Xujiahui Cathedral (Fig. 1) may have some similarity to Xiaoxishi-stone(Fig. 14) .

Recent surveys done in the Jingling Donglu, Shanghai show that stones like typical tuffs have also been used as Frame of Shikumen and decorative ornaments. Further researches are need to source this kind stones either for restoration or reconstruction.

## Conclusions

On the basis of a critical evaluation of the information gathered from previous recognition and geological data produced, it has been possible to determine the differences between three lithotypes. Meiyuanshi-stone is massive (no internal stratification), thick structureless, well sorted, and fine-grained trachytic tuff formed from fallout. Furthermore, the stone utilised in platform of the Baoguosi Temple has been identified as Meiyuanshi-stone. Xiaoxishi-stone is rhyolitic tuff which has an affinity on origin with Meiyuanshi-stone, but coarser in grain size and has layered structure. Dayinshi-stone is composed of both poorly sorted tuff and feldspathic sandstone.

The results provide not only a sound knowledge of the characteristics of the stone involved in monuments in Ningbo, but also a database of stone materials in neighbouring areas such as Shanghai. The preliminary examination of “grey stones” used for the construction of Xujiahui Cathedral (Fig. 1) may come from Ningbo. This expansion of the database will enhance our understanding in characterisation of monuments and potentially determine economic as well as cultural associations.

Today there are not database for the built historical natural stones in China. Further studies are needed to understand the natural stones used not only in Shanghai’s architectural heritage, but also for the construction and restoration of traditional Chinese gardens in Suzhou.

In terms of methodology and interpretation, cares should be taken when the stone samples are taken from monuments, which have been deteriorated or contaminated. Mineralogical and chemical composition might have been changed from fresh new stones to stones standing outside for decades or centuries.

## Data Availability

Not applicable.

## Abbreviations

ICP-MS: Inductively coupled plasma-mass spectrometry
XRD: X-ray diffraction
TAS: Total alkali-SiO_2_
LREE: Light rare earth elements
PDC: Pyroclastic density flow
